# Roles of Cellular Neighborhoods in Lung Cancer

**DOI:** 10.7150/jca.132848

**Published:** 2026-03-25

**Authors:** Rui Bai, Wenjie Sun

**Affiliations:** 1 Department of Pulmonary Oncology, Zhongnan Hospital of Wuhan University, Wuhan, Hubei China.; 2 Department of Radiation Oncology, The Affiliated Cancer Hospital of Zhengzhou University & Henan Cancer Hospital, Zhengzhou, Henan China.

**Keywords:** Cellular neighborhoods, Lung cancer, Spatial transcriptome, Immune microenvironment

## Abstract

The occurrence and development of lung cancer (LC) involve complex interactions between various cell types in the tumor microenvironment (TME). Understanding the spatial distribution and interaction mechanisms of these cells may be the key to overcoming LC. The advancements of single-cell and spatial transcriptome techniques have promoted our understanding of cellular neighborhoods (CNs) and their functions in the pathogenesis of LC. In this review, we focus on the impact of different etiologies on LC CNs and the current research status of CNs in LC. This review may provide new insights into the molecular mechanisms of LC pathogenesis, develop more refined classification principles for LC diagnosis, and offer new perspectives for LC treatment.

## Introduction

Thanks to the improvement of low-dose CT screening and advances in targeted therapy and immunotherapy, the mortality rate of lung cancer (LC) has significantly decreased in the past decade 1. But LC remains the leading cause of cancer death. Non-small cell lung cancer (NSCLC) is the main type of LC, accounting for approximately 80-85% of all LC cases 2. The development of LC is a heterogeneous and multi-step process involving multiple genetic and epigenetic changes which help tumor cells escape immune responses, promote cancer progression and metastasis 3.

Cellular neighborhoods (CNs) refer to the spatial relationships and interactions between specific cells and their surrounding cells in tissues or cell populations 4, 5. This concept is commonly used to describe the distribution pattern of cells in a specific microenvironment, emphasizing the interactions and functional connections between cells 6-8. Through CN analysis, researchers can better understand the complex structure and function of tissues 9, 10. For example, in the tumor microenvironment (TME), different types of immune cells, tumor cells and their interactions form specific neighborhoods that may affect tumor growth and metastasis 11, 12. CN analysis also helps identify intercellular signaling and interaction networks, which is crucial for understanding cancer progression. Because these interactions often determine the development and treatment response of cancer. A deeper understanding of the neighborhood relationship between immune cells and tumor cells in the TME can help develop new immune therapies.

The etiology of LC is still not fully understood, and the main pathogenic factors include smoking, occupational exposure, air pollution, ionizing radiation, pulmonary history, etc 13-17. When pathogenic factors disrupt the homeostasis of the lungs, it ultimately leads to LC. The composition of LC is not simply the accumulation of tumor cells, but a microenvironment composed of fibroblasts, immune cells, extracellular matrix, and microvessels 18, 19. There is a mutually restrictive relationship between tumor cells and the microenvironment 20. The interaction between cells is crucial at every stage of LC development 21, 22. Therefore, identifying LC-related CNs and understanding their functions are the key to deciphering the pathogenesis of LC. The advancement of technologies such as spatial transcriptome and single-cell sequencing has provided new avenues for studying CNs. Understanding the spatial environment and intercellular communication of cells in the microenvironment can reveal the complex pathogenesis of LC.

This article reviews the role of CNs in the pathogenesis of LC, analyzes the impact of different etiologies on CNs in LC, and the current research status of CNs in LC. This review describes the cell types involved in CNs of LC, the functions of CNs and their significance in the occurrence and development of LC. It also explores the key signaling pathways that may drive the formation of specific CNs. This review can improve our understanding of the pathogenesis of LC and provide new ideas for the treatment of LC by dissecting the CNs of LC.

## 1. Etiology-related cellular neighborhoods in LC (Figure 1)

### 1.1 Smoking

Smoking is associated with the onset of metabolic syndrome 23. Metabolic syndrome is generally accompanied by abnormalities in blood glucose, blood pressure, and blood lipids 24. Especially high blood glucose and abnormal blood lipids are related to the formation and progression of LC 25, 26. The changes in glucose metabolism and lipid metabolism mainly affect tumor associated macrophages (TAMs) 27. TAM, as one of the main cell types of TME, plays an important role in tumor progression through various mechanisms by secreting proteases, inflammatory mediators, growth factors, and disrupting anti-tumor immunity 28. Smoking not only increases the metabolism of LC cells, but also promotes the progression of LC by altering the immune regulatory function of TAMs 29. Studies showed that exposure to cigarette smoke extract (CSE) could induce macrophage M2 polarization. CSE also promoted macrophage to secrete vesicles containing circEML4. After uptaking by NSCLC cells, it interacted with intracellular ALKBH5 to promote m6A modification of SOX2, ultimately promoting NSCLC progression 30. In addition, the dysfunction of alveolar cells caused by smoking could promote the invasiveness of lung adenocarcinoma (LUAD).

Single-cell profiling of tumor specimens and distal normal lung tissue from 22 LUAD patients (13 non-smokers and 9 smokers) demonstrated that compared to normal lung tissue, tumor tissue had a lower proportion of natural killer cells (NK cells) and a higher proportion of B lymphocytes 31. Single-cell RNA sequencing (scRNA-seq) of normal lung cells adjacent to tumors showed that smoking led to upregulation of HLA-II and pro-inflammatory pathways (especially defense response to virus and response to type I interferon) in alveolar macrophages (AMs) 32. Another study utilized single-cell transcriptome and epigenetic sequencing to establish a single-cell multi-omics map of human lung tissue. The study elucidated the key roles of epithelial cells and immune cells in the evolution of LC, and identified LC susceptibility genes, providing precise diagnostic targets and key molecular phenotypes with potential clinical application value for subsequent drug development and intervention 33. An important study revealed how smoking drove the occurrence of early lung squamous cell carcinoma (LUSC) by disrupting the clonal dynamics of basal cells, providing a new perspective for understanding the “field cancerization” of LC 34.

### 1.2 Air pollution

Particulate matter (PM) is a variety of solid and liquid particles in the atmosphere, which has been identified as the main cause of LC 35. PM2.5 is a particle with aerodynamic size ≤ 2.5 μm, which can lead to an increase in the incidence rate and mortality of LC in the population 36. Engine exhaust and air pollution induce LC through genotoxicity, oxidative stress, and inflammation 37, 38. Research revealed that air pollution significantly promoted the accumulation of mutations in the LC genome, and the mutation load was dose-dependent with PM2.5 concentration, suggesting that air pollution was not only a mutagenic source of LC, but also a promoting factor for tumor development 39. A study found a new mechanism by which PM2.5 drove LC. PM2.5 did not directly cause cancer mutations in lung cells, but rather promoted the entry of a large number of macrophages into lung tissue, releasing IL-1β to create an inflammatory environment, leading to the malignant transformation of normal lung cells carrying carcinogenic mutations (EGFR or KRAS), thereby causing cancer 40.

### 1.3 Ionizing radiation

Ionizing radiation can induce the formation of immunogenic or immunosuppressive TME 41. If the balance shifts towards an immunogenic phenotype, radiation will cause tumor cells to release new antigens and damage associated molecular patterns (DAMPs) 42. These signals in turn lead to an increase in antigen presentation, thereby activating the innate immune system, increasing CD8+ cytotoxic T cell infiltration, and inhibiting immune suppressive cells 43. If the balance tilts towards the immunosuppressive phenotype, radiation will kill T lymphocytes in the TME, increase infiltration of bone marrow-derived suppressor cells (MDSCs) and regulatory T cells (Tregs) and promote the activation of cancer associated fibroblasts (CAFs), thereby promoting tumor growth 44, 45. Radiation not only has a regulatory effect on TME, but can also alter the immune characteristics of patients. As shown in the meta-analysis of different cancer types, patients experienced a systemic decrease in CD3+ and CD4+ peripheral T cells one month after the last radiotherapy 46. Another retrospective study evaluated the effect of RT on programmed cell death-ligand 1 (PD-L1) expression and CD8+ T cell infiltration in NSCLC patients. While PD-L1 expression in cancer cells showed no consistent trend, the density of CD8+ T cells increased after radiation 47. In addition, research showed that T cells, B cells, neutrophils and NK cells were enriched in the radiation sensitive group 48.

Radiation also reshape vascular system in TME. Radiation-induced endothelial cell dysfunction is characterized by increased permeability and apoptosis. Damage to vascular endothelial cells leads to increased expression of intercellular adhesion molecule (ICAM) and vascular cell adhesion molecule (VCAM), which play a key role in leukocyte migration and activation 49. Endothelial cell damage can also attract natural immune cells to the TME. Radiotherapy causes hypoxia by damaging blood vessels, and further exacerbates TME hypoxia by consuming oxygen and producing toxic substances 50. Hypoxia activates the hypoxia-inducible factor-1 (HIF-1) signaling pathway and promotes angiogenesis through vascular endothelial growth factor (VEGF) 51. Researchers analyzed single-cell data from NSCLC patients and found that macrophages with hypoxia had pro-angiogenic activity and may be potential therapeutic targets for NSCLC patients 52.

On the other hand, the activation of CAFs after radiation leads to changes in growth factor secretion and the release of numerous extracellular matrix (ECM) regulators which can promote angiogenesis, invasion, and metastasis of cancer cells 53. Research showed that alveolar epithelial cells type II underwent early apoptosis in direct response to ionizing radiation to clear damaged cells. Epithelial cell apoptosis destroyed the alveolar structure, causing the body to be unable to effectively clear damaged cells in a timely manner. At the same time, it was accompanied by a strong inflammatory response, exacerbating radiation-induced lung injury (RILI) and promoting the occurrence of LC 54.

### 1.4 Lung injury

Most NSCLC patients have a smoking history, which can lead to fibrosis and scar formation in lung tissue. In addition, diseases with increased pulmonary fibrosis, such as idiopathic pulmonary fibrosis (IPF), are associated with an increased incidence rate of LC 55, 56. Researchers used spatial single-cell proteomics to analyze 6 cases of IPF and 3 cases of normal lung samples, successfully identifying multiple cell types in lung tissue. Among them, CSMD1+ fibroblasts were enriched in fibrotic areas and co-localized with pro-fibrotic molecules such as TGF-β1 and CTHRC1. CD248+ fibroblasts existed in low inflammatory areas and were associated with the anti-fibrotic factor MGP 57.

To unravel the immune cell landscape of the lung and identify the mechanisms driving injury-associated immune pathology, spatial transcriptome analysis of lung tissues from healthy individuals and those with IPF was conducted, revealing that the majority of differentially expressed genes were located in the fibrotic region, with multiple chemokines highly expressed at the fibrotic edge and reduced expression levels in the alveoli. On the contrary, ECM related genes (COL1A1, COL1A2, and LUM) were lowly expressed at the edge of fibrosis and highly expressed in fibrosis and adjacent alveoli 58. Another study used spatial transcriptomics and the scRNA-seq to determine the unique cellular composition and localization of three disease-related niches. Fibrotic niche was composed of myofibroblasts and abnormal basal cells. Macrophage niche was located around the airway and adjacent to the airway lumen. Immune niche was characterized by obvious lymphocyte foci in fibrotic tissue. This spatial characterization of IPF niche will help identify drug targets and aid in the development of disease-related *in vitro* models 59. The study used image-based spatial transcriptomics techniques to explore the evolution of alveolar niche dysbiosis in IPF and identified 12 ecological niches with unique gene expression characteristics and cell type composition, including cell ecological niche C11 associated with macrophage accumulation in the airspace, with FABP4+ and SPP1+ two main populations. In the IPF sample, the distribution and proportion of these two types of macrophages changed with disease progression, indicating that the evolution of macrophage phenotype was related to the progression of IPF 60.

## 2. Cellular neighborhoods in LC

Tumor heterogeneity refers to the molecular biology or genetic changes that occur during the progression of tumors, resulting in differences in the growth rate, invasion ability, and drug sensitivity of different tumor cells 61, 62. It is a crucial characteristic in the process of tumor occurrence and development, a supporting point for tumor progression, and a key theory discovered through continuous exploration in recent years 63-65. Tumor heterogeneity not only affects diagnosis, but also has an impact on treatment, efficacy, disease monitoring, drug resistance, and prognosis 66, 67. The TME is the main source of heterogeneity in LC 68, 69. Therefore, in-depth analysis of the spatial landscape of the immune microenvironment in LC is crucial. Such analysis can aid in understanding its pathogenesis, screening disease biomarkers, and developing novel treatment strategies. With the application of technologies such as spatial transcriptomics and single-cell sequencing, we have gained a deeper understanding of the heterogeneity of CNs in LC 70.

### 2.1 Immune cell-related cellular neighborhoods (Figure 2)

#### 2.1.1 T cell-related cellular neighborhoods

T cells are an important component of the immune system in combating cancer, and the degree of T cell infiltration is positively correlated with the patient's treatment response and prognosis 71, 72. The spatial distribution of T cells in tumor tissue is closely related to the TME, and the microenvironment characteristics of different regions determine the functional status of T cells 73, 74.

CD8+ T cells promoted the infiltration of CD4+Foxp3+ Tregs and accelerated the growth of murine LUAD. This was achieved by increasing the levels of CCR5 chemokines in the TME in an IFN-γ and TNF-α dependent manner 75. A study used scRNA-seq to map the TME of NSCLC patients after receiving PD-1 combined chemotherapy, revealing that the CD4+ Tregs count was higher in the group that did not reach pathological remission, and the distribution of these cell types varied among the different groups. ZNF683+CD8+ tissue-resident memory T cells (Trm) and S100B+ NK cells were enriched in the non-pathological remission group, while GZMK+CD8+ effective memory T Cells (Tem) were enriched in the pathological remission group. Cell-chat analysis showed that in major pathological response (MPR) and non MPR tumors, CXCL12+ endothelial cells strongly interacted with CD8+ Trm and CD8+ Tem cells, and might participate in lymphocyte migration through the CXC12-CXCR4 axis in the MPR group. The study also found that CLEC9A+ cDC1 and CCL22+ mDC were highly correlated with GZMK+CD8+ Tem cells through specific ligand receptor interactions 76. These observations provide a deeper understanding of how the cellular niches influence T cell functions in LC.

#### 2.1.2 Macrophage cell-related cellular neighborhoods

TAMs have multiple functions, including promoting tumor growth, invasion, metastasis, and immune escape, and their functional diversity reflects the heterogeneity of cells 77, 78. The functional diversity of TAMs is not only influenced by their origin, but also regulated by local factors such as tumor type, organs, and microenvironment 79, 80.

The results of single-cell sequencing showed that macrophages were the most common cell population in the immune microenvironment of myeloid cells and macrophages with CD163 positivity had the highest enrichment level in solid tumors. In addition, CD163+ macrophages were strongly correlated with Treg and CD8+ T cells, indicating a potential interaction between macrophages and T cell populations in the immune microenvironment of NSCLC 81. The spatial transcriptome data revealed that tumor cells interact with SPP1+ macrophages and COL11A1+ CAFs, promoting the deposition and entanglement of collagen fibers at the tumor boundary, hindering T cell infiltration, and leading to adverse outcomes 82. By using scRNA-seq to analyze macrophages in NSCLC tissues, further investigation found that they could be subdivided into seven main subgroups, including two monocyte macrophage subgroups and five macrophage subgroups. Each subgroups had different inflammation related gene expression characteristics. Among them, IL1B+and FCN1+ subgroups were more abundant in adjacent cancer tissues, while LGMN+ and APOE+ subgroups had a higher degree of infiltration in tumor tissues. Macrophages derived from tumor tissue exhibit an anti-inflammatory phenotype and are associated with poorer progression free survival (PFS) in patients 83.

In addition, research showed that pseudomonas aeruginosa promoted the secretion of CXCL9 by TAMs and enhances CD8+ T cell activity, thereby enhancing the immune response of NSCLC to anti-PD-1 therapy 84. Understanding the mechanism of immune suppression mediated by macrophages can develop the targeted intervention strategies aimed at macrophages, thereby enhancing the efficacy of LC immunotherapy.

#### 2.1.3 Tertiary lymphoid structures

There are different states of tertiary lymphoid structures (TLSs) in the TME, among which activated TLSs are associated with good prognosis, while the hypoxic microenvironment inhibits the development of TLSs and is associated with poor prognosis in LC 85-87.

Single-cell sequencing of NSCLC patients receiving PD-1 combined chemotherapy identified several unique subtypes of memory B cells: IGHG4+ plasma cells, SCGB3A1+, IFITM3+, RBFOX2+ memory B cells. Based on the TLSs gene set, TLS scores were calculated for each type of B cell. The TLS score in tumors was significantly higher than that in adjacent normal tissues, especially in MPR tumors. The highest TLS scores were observed in IFITM3+ memory B cells, HLA-DQA2+ memory B cells and RBFOX2+ memory B cells, indicating their predominance within TLSs 76. Through spatial transcriptome analysis, the study further revealed the close correlation between TLS distribution and microenvironment status. The activation level of TLS was significantly reduced in low oxygen regions, while TLS exhibited higher maturity in invasion boundary regions with sufficient oxygen supply 82. A study conducted a combined analysis of scRNA-seq and spatial transcriptomics on tumor tissue samples from 12 NSCLC patients before and after neoadjuvant immunotherapy combined with chemotherapy. Research found that treatment significantly enhanced the infiltration of immune cells such as T/NK cells and plasma cells and increased fibroblast activity. The formation of TLS was observed in patients with pathological remission, suggesting that enhanced immune response and spatial tissue remodeling were closely related to treatment efficacy 88. These findings underscore the function of TLSs in LC.

#### 2.1.4 Cancer associated fibroblast-related cellular neighborhoods

CAFs play multiple key functions in the TME, interacting with malignant cells, immune cells and other stromal cells through various mechanisms and have a significant impact on the occurrence, development, metastasis and therapy efficacy of tumors 89, 90. Research found that CAFs promoted glycolysis in non-neuroendocrine (non-NE) small cell lung cancer cells (SCLC), thereby activating STING signaling in T cells and promoting chemokine expression. On the other hand, non-NE SCLC cells promoted the presence of antigen presenting CAFs, which might contribute to CD8+ T cell recruitment and Treg differentiation 91.

Through scRNA-seq and spatial transcriptome analysis of NSCLC tumor tissue, it was found that there was an increase in Th17 cells and inflammatory fibroblasts, and a decrease in Treg cells and stromal fibroblasts in effectively treated patients. SELENOP+ macrophages aggregated at tumor boundaries and lymphatic structures, synergistically recruiting T cells with antigen-presenting fibroblasts. After treatment, intercellular communication was enhanced, and cholesterol and other pathways were activated to promote immune response 88. Another single-cell sequencing was performed on tumor samples of NSCLC patients before and after anti-PD-1 combined chemotherapy. Based on pathological evaluation, patients were divided into responders and non-responders. Research found that after combination therapy, the malignant epithelial cells in the tumor almost completely disappeared in responders, while non-responders had more monocytes/macrophages and dendritic cells, as well as a significantly increased proportion of COL11A1+ CAFs 82.

Additionally, scRNA-seq was performed on tumor, paired tumor adjacent, and normal samples from 16 NSCLC patients. It was found that POSTN+ CAFs and SPP1+macrophages had tight localization and might promote ECM remodeling and immunosuppressive TME formation 92. Interestingly, the research team explored the spatial localization of fibroblasts relative to other cells in the lung TME and found that adjacent cells were cancer cells, macrophages, fibroblasts, and endothelial cells in order from near to far, indicating that fibroblasts and cancer cells could directly interact with each other 93. These findings emphasize the controversial roles of CAFs and further analysis is required to figure out their contributions to LC progression.

### 2.2 Tumor cell-related cellular neighborhoods (Figure 3)

Tumor cells work together with neighboring cells to promote disease progression through interactions, signal transduction and physical contact. scRNA-seq and spatial transcriptome analysis revealed enrichment of CXCL14+ myofibroblasts CAF (myCAF) in advanced LUAD. This subgroup enhanced tumor invasiveness through epithelial to mesenchymal transition and angiogenesis and endowed LUAD cells with resistance to EGFR-TKI 94. TKI treatment could lead to immunogenic cell death (ICD) and release of high mobility group box 1 protein (HMGB1) in LC cells. HMGB1 promoted macrophages to release cytokines and recruit T cells. At the same time, HMGB1 derived from tumor cells could also activate the NF-κB signaling pathway in T cells, leading to an increase in cytotoxic T lymphocyte-associated protein 4 (CTLA-4) expression, which weakened T cell function and created an immunosuppressive microenvironment 95.

Other studies also showed the functions of some molecules. The high expression of JAG2 in NSCLC was negatively correlated with survival rate. Inhibiting the expression of JAG2 induced the expression of ligand DLL1/4 in NSCLC cells, activated the notch signaling pathway in macrophages, enhanced macrophage immune stimulatory function and triggered T-cell dependent anti-tumor immunity 96. SOX2 promoted the secretion of C-C motif chemokine ligand 2 (CCL2) by NSCLC cells, leading to increased recruitment of Tregs and inhibition of CD8+ T cell infiltration 97. NSCLC cells transferred extracellular vesicles containing β-TrCP to CD8+ T cells, promoting the ubiquitination of YAP1 protein and inhibiting the transcription and expression of downstream genes, leading to the inactivation of mTORC1 signaling pathway in CD8+ T cells and ultimately causing CD8+ T cell exhaustion 98.

Space transcriptome technology provides a new perspective for understanding. For example, the interaction between B cells and cancer cells was more frequent in LUAD, while the interaction between neutrophils, cancer cells, and macrophages was more common in LUSC. By analyzing the CNs, the research team found that the TME of LUAD and LUSC had different spatial structures. The tumor boundary (CN1) was the most abundant in LUAD, while the tumor compartment (CN7) was the most abundant in LUSC, reflecting the immunological rejection characteristics of LUSC. The CN enriched with macrophages (CN3) in LUAD was associated with poor survival rates, indicating that the spatial dynamics of macrophages may play an important role in influencing patient prognosis 99.

## 3. Key signaling pathways in shaping cellular neighborhoods (Figure 4)

### 3.1 Metabolites

The characteristics of TME are local competition for nutrients, accumulation of metabolic waste, and unfavorable pH values. The common demand for similar nutrients among various cells within the TME leads to potential competitive TME and immune suppression. And metabolic pathways fundamentally participate in the determination of cell fate and cellular programs 100, 101. Glucose metabolism, lipid metabolism, and amino acid metabolism are the main components of tumor cell metabolism.

High expression of N-myc downstream-regulated gene 1 (NDRG1) in LUAD stabilized lactate dehydrogenase A (LDHA) by inhibiting its ubiquitination, thereby enhancing glycolysis and promoting lactate accumulation. This process promoted immune suppression by inducing M2 macrophage polarization and impairing the function of CD8+ T cells 102. The inactivation of STK11 in LUAD affected the lactate transporter monocarboxylate transporter 4 (MCT4), leading to increased lactate production and efflux in the TME. Excessive lactate could polarize macrophages towards M2 type, thereby reducing T cell infiltration and inhibiting their function 103. In addition, increased glycolytic activity in NSCLC patients after radiotherapy could lead to lactate accumulation and TME acidification 41.

Research also showed that TME could also provide lipids to cancer cells. The high lipid levels in TME altered the function of immune cells, with a large amount of free fatty acids leading to lipid accumulation in NK cells, thereby reducing their immune response. Immunosuppressed Tregs and TAMs also depend on fatty acid oxidation 104, 105. LC cells secreted granulocyte-macrophage colony-stimulating factor (GM-CSF) to activate the PPAR-γ of TAM, promoting the release of fatty acids and cholesterol to support tumor metabolic needs 106. Targeting lipid metabolism could inhibit the PI3K/AKT/mTOR signaling pathway, downregulating fatty acid synthesis and thereby inhibiting fatty acid oxidation in TAMs. On the other hand, it promoted the secretion of CXCL10 and CXCL11, thereby increasing the infiltration of CD8+T cells, ultimately inhibiting tumor progression 107.

In the immune microenvironment of LC, tumor cells compete with immune cells for the uptake of glutamine and glucose. Each cell type has a preference for the uptake of glucose and glutamine. Compared with T cells, LC cells are more likely to take up glutamine. Inhibiting TAM's uptake of glutamine can inhibit its transformation into M2-like macrophages 108.

### 3.2 Hypoxia

Hypoxia combined with high levels of ROS causes a chronic inflammatory state and suppresses the infiltration of immune cells such as CD8+ T cells, CD4+ T cells, NK cells and DCs. In addition, this condition simultaneously increases the aggregation of immunosuppressive cells, such as Tregs and MDSCs, ultimately leading to an immunosuppressive environment 109. The activation process of HIF-1α has stage specific characteristics. Under mild hypoxia, HIF-1α exhibits partial transcriptional activity and enhances primary adaptive responses such as glycolysis and immunosuppression. As oxygen tension further decreases to moderate hypoxia levels, HIF-1α is fully activated, significantly inducing stronger responses such as angiogenesis 110. Under severe hypoxia, fully activated HIF-1α continues to accumulate to high levels, causing a significant accumulation of lactate and extracellular acidification, ultimately triggering pathological outcomes such as programmed necrosis. Under acute hypoxia, neutrophils promote degranulation and glycogen storage, and exacerbate inflammatory damage. Macrophages promote IL-1β production and their migration ability. DCs secrete more pro-inflammatory cytokines. Under chronic hypoxia, tumor cells promote PD-L1 expression and inhibit the function of T cells. The upregulation of apoptotic gene expression and decreased migration ability in DCs lead to tumor immune escape. TAMs enhance glycolysis and lipid uptake, supporting their immunosuppressive function. Chronic hypoxia also weakens neutrophils function through DNA hypermethylation 111. Hypoxia induces NSCLC cells to secrete extracellular vesicles, promoting macrophages M2 polarization, and resulting in NSCLC metastasis 112. Hypoxia also promotes the production of HIF-1α in NSCLC TME, which can activate immunosuppressive genes and pro-tumor genes 113. It can also recruit Tregs and MDSCs, creating an environment conducive to tumor growth 114.

Single-cell sequencing analysis was performed on NSCLC. The results showed that compared with low-grade tissue subtypes, the interaction between neutrophils, endothelial cells and cancer cells was significantly enhanced in high-grade tumors. It was also observed that the proportion of HIF-1α+ neutrophils was negatively correlated with patient survival prognosis 81. The study also found that in the hypoxic areas of NSCLC, especially in the necrotic areas, a large number of macrophages with high HIF-1α expression were found, which promoted the transcription of the angiogenesis-related gene VEGF. HIF-1α+ TAMs promoted tumor angiogenesis, increased the density of tumor blood vessels and facilitated local invasion of tumors 115.

### 3.3 Angiogenesis

Tumor associated vasculature (TAV) has characteristics such as structural distortion, strong leakage and disrupted blood flow, causing hypoxia and acidosis in the TME. These abnormalities not only hinder the infiltration of cytotoxic T lymphocytes (CTLs), but also inhibit their survival and function, thereby promoting tumor metastasis and becoming the main barrier for immunotherapy 116.

Spatial transcriptome analysis showed that although VEGFA and VEGFB were found to be expressed in both LUAD and LUSC, their receptors were more common in LUAD, especially in fibroblasts 117. Single-cell sequencing results showed significant spatial heterogeneity in LUAD. CD4+ primitive T cells in the central region of the tumor had a stronger ability to promote angiogenesis. In contrast, CD4+ primitive T cells in the peripheral region of the tumor had stronger cytokine production and pro-inflammatory ability. The signaling pathways that promoted angiogenesis, fatty acid synthesis and hypoxia were significantly enriched in macrophages in the central region of the tumor compared to macrophages in the peripheral region 118.

Another study identified subpopulations of tissue-resident neutrophils (TRNs) in NSCLC TME. The results showed that tumor associated neutrophils (TANs) and adjacent normal tissue associated neutrophils (NANs) had different expression profiles and neutrophils were the main source of VEGFA expression in the TME, confirming the important pro-angiogenic role of TRNs 119. To explore potential intercellular communication, researchers conducted single-cell transcriptomic analysis to obtain intercellular signaling network. In the VEGF signaling network, as LUSC progressed, plasmacytoid DCs and monocyte-derived macrophages (mo-macs) replaced non-classical monocytes. In addition, mo-macs and plasmacytoid DCs played a dominant role in the TGF-β and CXCL signaling networks, which were associated with EMT, angiogenesis and immune suppression 120.

## 4. Conclusion

The rapid developments of single-cell and spatial transcriptome techniques have revealed the tumor heterogeneity and immune microenvironment dynamics of LC, elucidated the communication network of CNs in the LC microenvironment, significantly enhancing our understanding of the pathogenesis of LC, and providing an important basis for precise classification and treatment strategies of LC.

Although there have been many studies focused on conquering LC, it remains one of the malignant tumors with the highest mortality rate. Given the complexity of the TME, interactions between different types of cells jointly regulate tumor progression. A deeper understanding of CNs may provide new treatment options. With the advancements of single-cell and spatial transcriptome technologies, we can also better understand the molecular mechanisms underlying LC formation.

With the increasing variety and precision of detection technologies, an increasing number of tumor biomarkers are being detected. However, there is still a lack of reliable biomarkers to predict patient prognosis and treatment response. The spatial heterogeneity of tumors can lead to different clinical outcomes. By mining local cell interaction patterns and relative spatial positions of cells, CNs can stratify patient prognosis and improve the accuracy of predicting treatment efficiency. Although CNs provide us with a more cutting-edge perspective for a deeper understanding of LC, further in *vitro* experiments and animal models are still needed to explore specific molecular mechanisms. Understanding the biological and spatial characteristics of LC can provide us with a deeper understanding of the tumor ecosystem and ultimately offer new strategies for spatially targeted therapy.

Future research can focus on potential molecular mechanisms related to CNs formation and higher-level organizational structure formation. The successful progress of these studies is expected to elucidate the function and clinical significance of CNs, provide a more solid theoretical basis for identifying early LC, detecting LC-related biomarkers and evaluating LC prognosis.

## Figures and Tables

**Figure 1:**
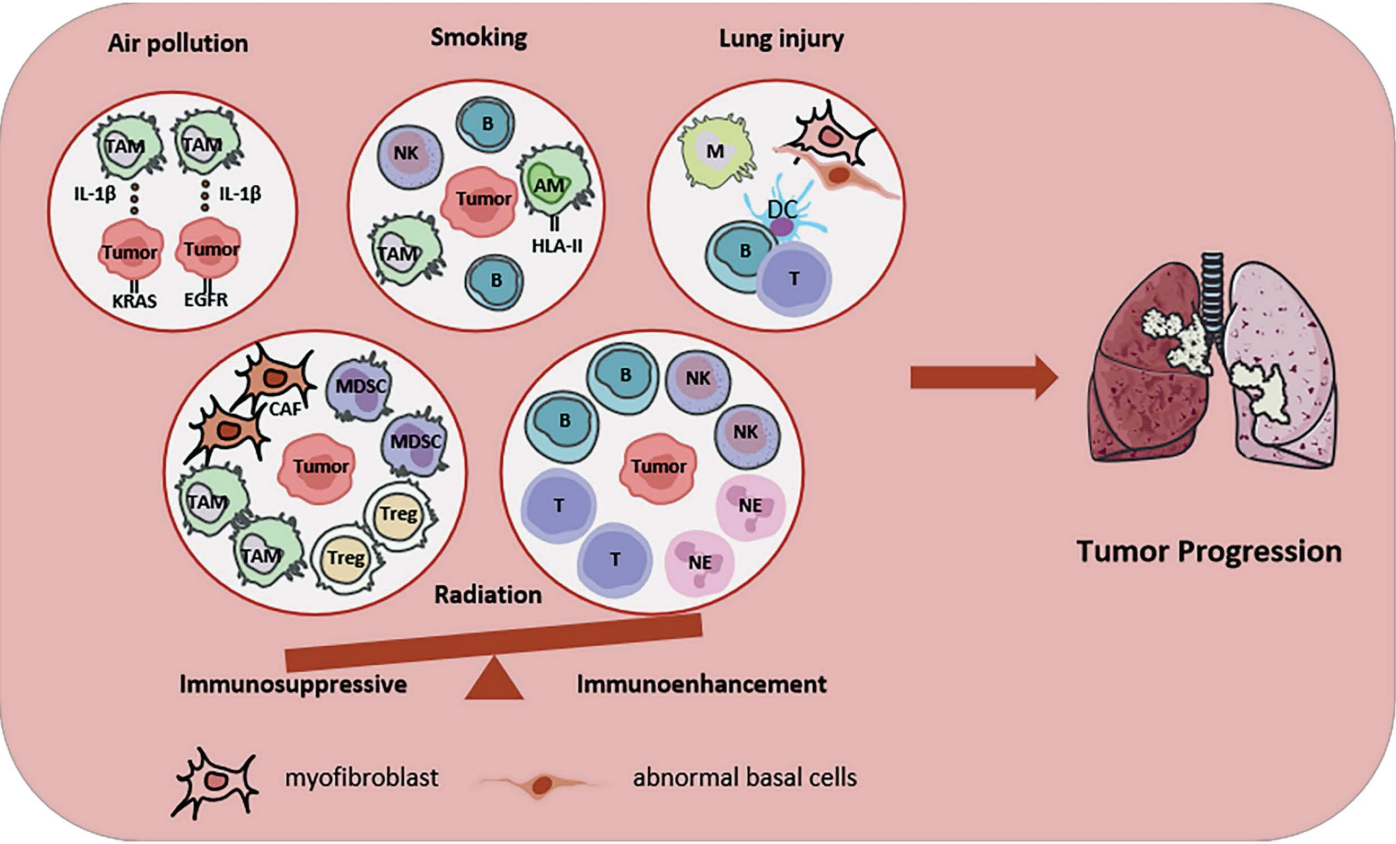
Etiology-related CNs in LC. Etiology-related CNs, such as smoking, air pollution, ionizing radiation and lung injury, are shown. Air pollution promotes IL-1β secretion of macrophage, induces the malignant transformation of normal lung cells carrying carcinogenic mutations (EGFR or KRAS). Smoking promotes the aggregation of NK cells, B cells and TAM in LC CN, as well as the expression of HLA in AM. Lung injury promotes the formation of three types of niches. One enriches macrophages, one enriches DCs, B cells and T cells, and another enriches myofibroblast and abnormal basal cells. Radiotherapy regulates the immune balance of LC. Immunosuppressive CNs enrichment CAFs, MDSCs, TAMs and Tregs. Immunoenhancement CNs enrichment B cells, T cells, NK cells and neutrophils. All the above pathological factors will promote LC progression to some extent. Abbreviations: TAM, tumor associated macrophage; CAF, cancer associated fibroblast; MDSC, bone marrow-derived suppressor cell; NE, neutrophil; NK, natural killer; AM, alveolar macrophage; DC, dendritic cell; Treg, regulatory T cell.

**Figure 2:**
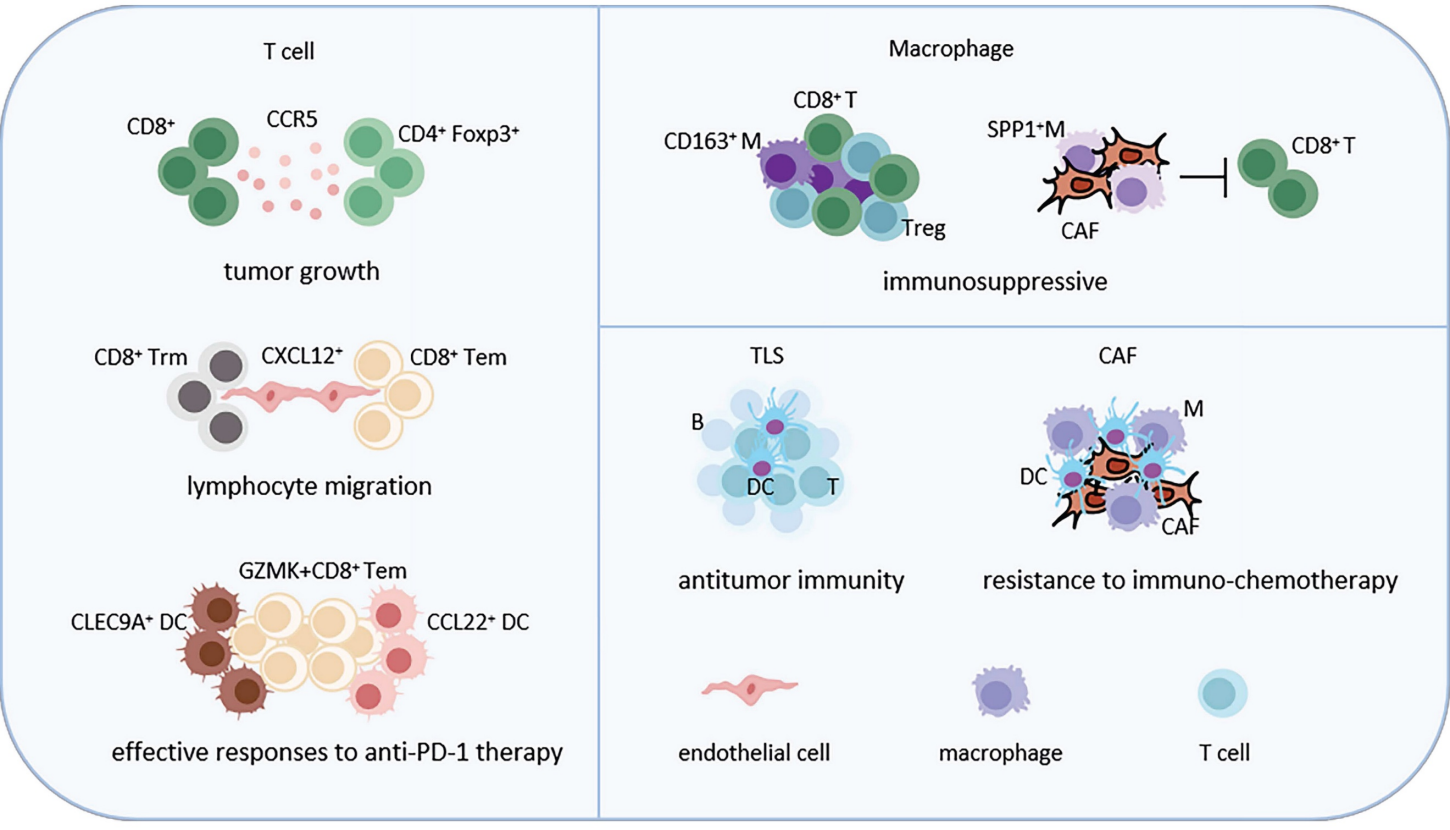
Representative immune cell-related CNs in LC. T cell-associated CNs, macrophage-related CNs, TLSs and cancer associated fibroblast-related CNs, reprogramming of the TME and interactions with neighboring cells, are shown. Abbreviations: Trm, tissue-resident memory T cells; Tem, effective memory T Cells; DC, dendritic cell; CAF, cancer associated fibroblast; TLS, tertiary lymphoid structure; Treg, regulatory T cell.

**Figure 3:**
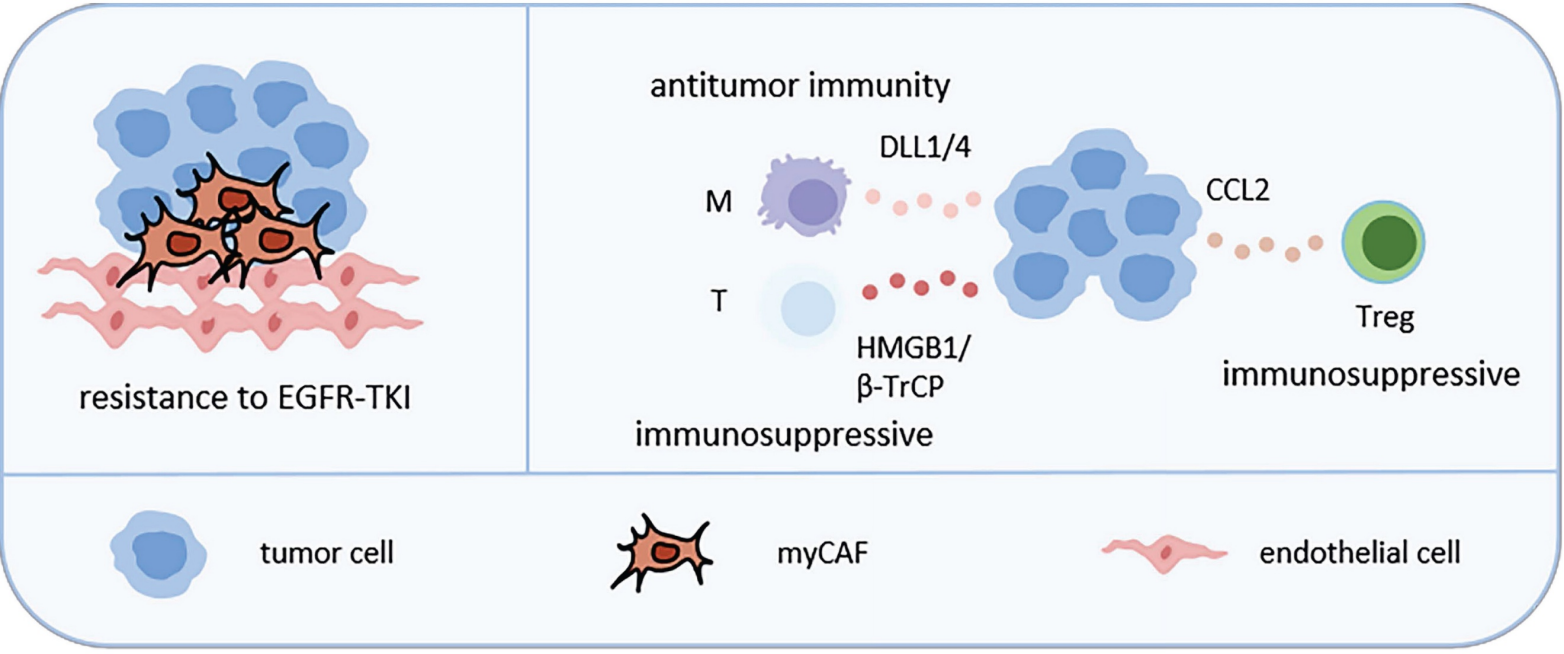
Representative tumor cell-related CNs in LC. CAFs can interact with LC cells to promote their invasiveness and resistance to EGFR-TKI. Tumor cells can attract Treg by secreting CCL2, attract macrophages by secreting DLL1/4, attract T cells by secreting HMGB1 or β-TrCP, thereby reshaping TME. Abbreviations: myCAF, myofibroblasts cancer associated fibroblast; NE: neutrophil; M: macrophage; LUAD: lung adenocarcinoma; LUSC: lung squamous cell carcinoma; Treg, regulatory T cell.

**Figure 4:**
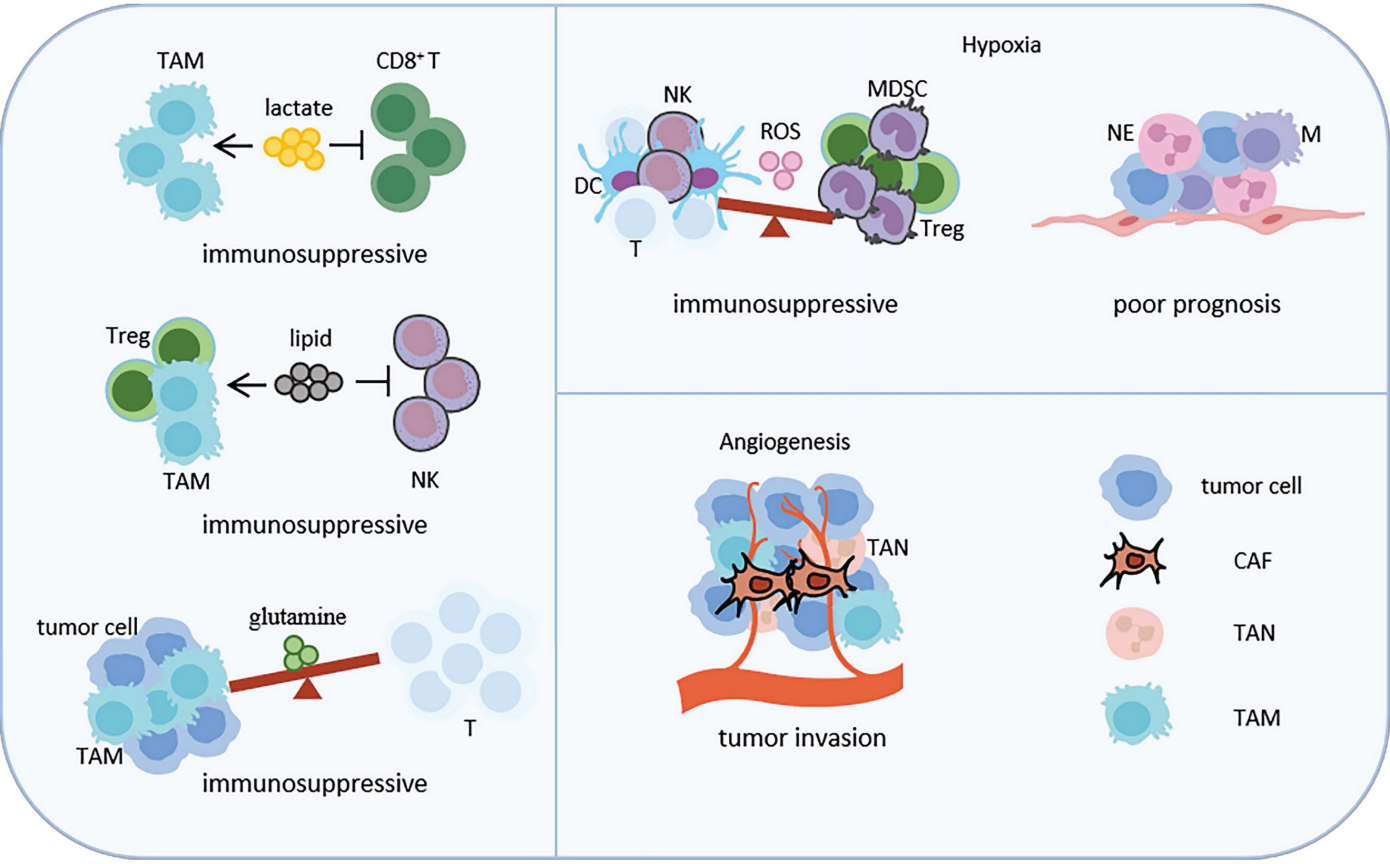
Representative key signaling pathways in shaping CNs in LC. Key signaling pathways-related CNs, such as metabolite, hypoxia and angiogenesis, are shown. Lactic acid promotes TAM infiltration and inhibits CD8+ T cell infiltration. Lipids promote TAM and Treg aggregation and inhibits NK cells aggregation. Tumor cells and TAMs compete to utilize glutamine, inhibiting the nutritional metabolism of T cells. These three metabolisms lead to the formation of an immunosuppressive microenvironment. Hypoxia promotes the enrichment of Treg and MDSC, and inhibits the enrichment of NK cells, T cells and DC by regulating the content of ROS, leading to immunosuppression. In addition, hypoxia also promotes the interaction between neutrophils, macrophages, LC cells and endothelial cells, promoting the invasiveness of tumors. Angiogenesis promotes infiltration of TAM, TAN and CAF, resulting tumor invasion. Abbreviations: TAM, tumor associated macrophage; CAF, cancer associated fibroblast; MDSC, bone marrow-derived suppressor cell; NE, neutrophil; NK, natural killer; DC, dendritic cell; TAN, tumor associated neutrophil.
